# A new way to contemplate Darwin's tangled bank: how DNA barcodes are reconnecting biodiversity science and biomonitoring

**DOI:** 10.1098/rstb.2015.0330

**Published:** 2016-09-05

**Authors:** Mehrdad Hajibabaei, Donald J. Baird, Nicole A. Fahner, Robert Beiko, G. Brian Golding

**Affiliations:** 1 Centre for Biodiversity Genomics @ Biodiversity Institute of Ontario and Department of Integrative Biology, University of Guelph, Ontario, Canada N1G 2W1; 2 Environment and Climate Change Canada @ Canadian Rivers Institute, University of New Brunswick, 10 Bailey Drive, PO Box 4400, Fredericton, New Brunswick, Canada E3B 5A3; 3 Faculty of Computer Science, Dalhousie University, 6050 University Avenue, PO Box 15000, Halifax, Nova Scotia, Canada; 4 Department of Biology, McMaster University, 1280 Main Street West, Hamilton, Ontario, Canada L8S 4K1

**Keywords:** DNA barcoding, phylogenetics, genomics, biodiversity, taxonomy, environment

## Abstract

Encompassing the breadth of biodiversity in biomonitoring programmes has been frustrated by an inability to simultaneously identify large numbers of species accurately and in a timely fashion. Biomonitoring infers the state of an ecosystem from samples collected and identified using the best available taxonomic knowledge. The advent of DNA barcoding has now given way to the extraction of bulk DNA from mixed samples of organisms in environmental samples through the development of high-throughput sequencing (HTS). This DNA metabarcoding approach allows an unprecedented view of the true breadth and depth of biodiversity, but its adoption poses two important challenges. First, bioinformatics techniques must simultaneously perform complex analyses of large datasets and translate the results of these analyses to a range of users. Second, the insights gained from HTS need to be amalgamated with concepts such as Linnaean taxonomy and indicator species, which are less comprehensive but more intuitive. It is clear that we are moving beyond proof-of-concept studies to address the challenge of implementation of this new approach for environmental monitoring and regulation. Interpreting Darwin's ‘tangled bank’ through a DNA lens is now a reality, but the question remains: how can this information be generated and used reliably, and how does it relate to accepted norms in ecosystem study?

This article is part of the themed issue ‘From DNA barcodes to biomes’.

It is interesting to contemplate a tangled bank, clothed with many plants of many kinds, with birds singing on the bushes, with various insects flitting about, and with worms crawling through the damp earth, and to reflect that these elaborately constructed forms, so different from each other, and dependent upon each other in so complex a manner, have all been produced by laws acting around us.

—Charles Darwin [1]

## 1. Introduction: challenges of biodiversity monitoring

### (a) Level of organization

Biologists' fascination with elaborating the sheer variety of species inhabiting the tangled bank alluded to in the closing paragraph of Darwin's *On the origin of species* continues apace, through the practice of what is now referred to as biodiversity science. An umbrella term for biological diversity, biodiversity has been defined succinctly by E.O. Wilson as ‘in one sense, everything’ [2]. This bold definition reminds scientists that they should not shy away from studying all aspects of biodiversity from biomolecules to biomes. The reality is, of course, that due to limitations in breadth of taxonomic expertise, coupled with a lack of an all-encompassing observation method, biodiversity has been studied in a circumscribed fashion thus limiting our ability to develop universal theories and practice in this critical area of science. The field biologist–taxonomist axis is the prime generator of biodiversity information. Their specialization on specific phylogenetic groups results in idiosyncratic knowledge generation, frustrating our ability to test general theories. Although the most common unit of taxonomic inquiry is the species, there is no consensus set of criteria for defining a species, nor can there be [3,4]. At the level of populations, species are studied and their characteristics and spatio-temporal distributions are investigated in various scenarios including conservation management, epidemiology or mapping invasive and harmful organisms. Multispecies assemblages occupying a habitat or ecosystem are targets of biodiversity analysis, and their functional roles and dynamics in space and time can be linked to environmental changes such as an altered climate or other anthropogenic or natural disturbances. However, due to difficulties in taxonomic identification, models of ecological change suffer from an inability, mainly through coarse morphological examination, to consistently, reliably and accurately measure taxonomic changes. Furthermore, it has been shown that biodiversity analysis at family or genus level may not provide sufficient information for capturing changes to ecosystem status [5,6].

### (b) Inferring ecosystem state from biodiversity information

Aside from the level of biological organization targeted for biodiversity analysis, inquiries have been limited due to differing levels of comprehension. Perhaps, the most relevant example is the concept of ‘bioindicator’ species where ecological conditions or status are determined by comparative analyses of pre-identified tolerant or sensitive taxa [7]. For example, benthic macroinvertebrates have been used as bioindicators of aquatic ecosystems. It has been argued that differential sensitivity of these organisms to environmental perturbations, which can result in changes to their communities, can support their use as indicators of ecosystem status. Essentially, biodiversity information for these selected taxa is the basis for comparative biological analyses of whole-ecosystem status (e.g. biomonitoring). Although the use of bioindicators has provided much-needed direct biological data in ecological and environmental status analysis, the fact that whole-ecosystem status is inferred through analyses of a small subset of taxa could miss critical changes in unobserved parts of the ecosystem. Additionally, in order to assess ecological processes and their linkages to biodiversity, it is important to be able to consider biodiversity of all relevant groups of organisms and their interactions [8]. An ability to observe biodiversity across its full phylogenetic breadth offers real potential to study higher-order ecological processes and structures by connecting and interpreting the occurrences of groups of organisms that until now have proved difficult to consistently observe and identify.

### (c) The biomonitoring bottleneck

Another important consideration for biodiversity analysis is the capacity to generate biodiversity information with appropriate frequency so that the data can support a monitoring scenario. Even if conventional identification approaches (e.g. morphological examinations) are appropriate for a group of taxa, it is important to consider the effort and time required to identify biodiversity at a given site versus the frequency required to accurately monitor biodiversity at that site. This issue poses a unique challenge and may require the consideration of trade-offs (see below). Additionally, the biomonitoring regime could vary depending on the habitat being sampled as well as method of inquiry. Systematic attempts at measuring the effort and time required for identifying certain groups of biota in habitats such as tropical forests attest to difficulty in operationalizing biomonitoring programmes. For example, the sampling and identification time required for 15 different taxa at five tropical sites was estimated as 18 200 person-hours [9,10]. Even in temperate regions, countries are struggling to implement biomonitoring plans and many sites are analysed only once or in low frequency (see below).

**Figure 1.**
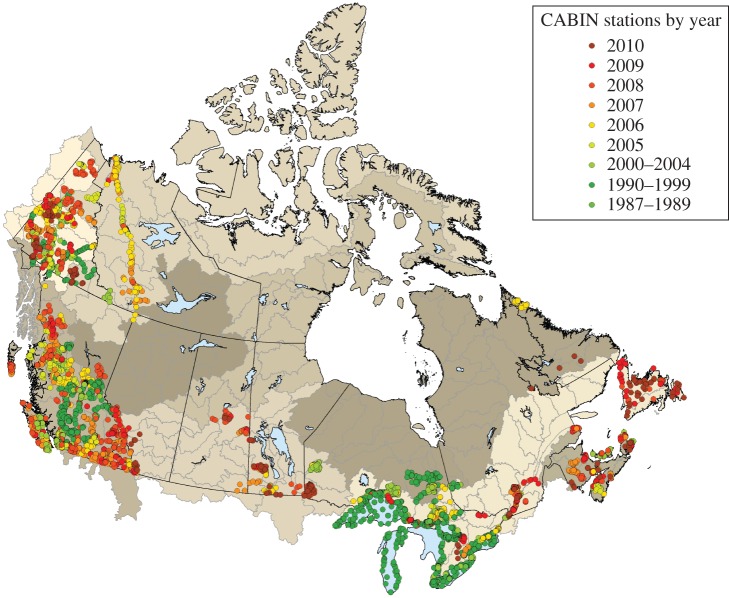
A map of Canadian watersheds, indicating the current spatial and temporal coverage of 5277 biomonitoring sites studied by the Canadian Aquatic Biomonitoring Network (CABIN) visited between 1987 and 2010. The distribution of sites indicates major gaps in spatial coverage related to the geographical scale of the country and its high level of remoteness.

Sample processing for taxonomic analysis is also constrained by the taxonomic competency of the operator. While this can be supplemented by gaining access to outside help from taxonomic experts, it is also constrained by cost and availability. It has long been recognized that scaling up biomonitoring programmes for regional or national assessment is often not achievable where taxonomic knowledge is poor or expertise is lacking. This situation is particularly problematic for areas where sites are far from population centres, or are difficult to access. For example, the Canadian Aquatic Biomonitoring Network has the distinction of being the world's largest consistently observed continental-scale national biomonitoring network in current operation. Yet despite significant effort and investment from its partners, the network contains many gaps in coverage at national scale (figure 1). In addition to the cost of accessing remote sites, a significant obstacle to achieving the critical data coverage necessary for national reporting on river health is the sheer numbers of samples that would be required to be processed for taxonomic analysis. With a shortage of taxonomic expertise [11], and the inevitable high costs of time-consuming, microscopy-based analysis, it is difficult to see how this network could realistically expand its coverage to permit true national-scale reporting based on up-to-date information.

## 2. Molecular systematics and the DNA barcode paradigm

Systematic biology has contributed immensely to our understanding of biodiversity. For example, phylogenetic analyses aim at reconstructing evolutionary relationships of a set of taxa through comparative analysis of characteristics shared by their evolutionary history (e.g. synapomorphies). Phylogenetic analyses have become more popular because of the availability of genetic information (as characters), which has triggered a concerted effort to reconstruct the Tree of Life for major groups of organisms [12]. Consequently, genetic and phylogenetic information are now an integral part of most biodiversity studies. However, most phylogenetic analyses, especially in eukaryotes, target evolutionary lineages at higher taxonomic levels (e.g. order, family), and sampling regimes usually include representative taxa for a given lineage. Efforts have been made to increase the statistical confidence of phylogenetic studies by sampling more taxa and increasing the number and diversity of genetic information (i.e. ‘phylogenomics’, [13]). This trend has continued to grow and we are witnessing a more elaborate linkage between phylogenetic reconstructions and addressing evolutionary questions.

In contrast with DNA-based phylogenetic investigations where evolutionary relationships are the focus of analysis, DNA barcoding has taken advantage of comparative sequence analysis for identifying specimens to the species level [14]. By focusing investigations on one or a few gene regions, DNA barcoding enables identification of unknown specimens [15]. DNA barcoding has gained momentum and has been applied for almost all groups of organisms from mammals to microbes [16]. These investigations have also illuminated some of the limitations of using a minimalistic DNA-based approach to identifying species. The species concept is heavily debated and any methodology used in identifying species will undoubtedly be impacted by this conceptual uncertainty. However, given the utility of identifying species as the ‘first step’ in addressing a wide range of biological questions, DNA barcoding has provided a much-needed solution for many research questions as well as socio-economic applications. Additionally, patterns of genetic divergence among unidentified specimens as compared to identified taxa have provided a powerful means for investigating new and cryptic species [17].

## 3. DNA barcodes for ecological inference

Currently, biological observation of macrofauna is constrained by data quality, particularly relating to the ‘lowest taxonomic level’ problem. Even when taxonomic keys are available to identify organisms within particular groups, they are often misinterpreted, incomplete or employed by users with limited training. While programmes are in place to provide quality assurance for identification for many purposes, these are not consistently employed to any significant degree in field-based research. Moreover, few standards exist in terms of how samples should be processed. When researchers state that organisms in the sample were identified to a specified taxonomic level (e.g. genus or species), what they generally mean is these organisms were identified to the best of our ability, based on the assumption that the keys available covered all the material being processed. Moreover, the fact that many organisms cannot be identified beyond a much higher taxonomic level (e.g. order) may be recorded, but often is not even mentioned. The consequence is that within certain areas of research, we have become comfortable with a technique that provides incomplete information. Orlofske & Baird [18] highlighted this problem of taxonomic sufficiency in relation to river benthos by showing that in a typical benthic sample collected in an area where familiarity with the local fauna was good, it was not possible to confidently identify more than 50% of the larval specimens of four commonly occurring insect orders to genus level. This was due to the presence of many early larval–stage specimens which lacked the distinguishable characters necessary for genus-level identification by a team of taxonomic experts. While this may come as no surprise to river scientists, it is nonetheless an unstated reality that faces sample identification: the current method is fundamentally flawed in that many taxa may be listed as falsely missing simply because they lack distinguishing characters at the time of sampling. This limitation also applies to cryptic species within commonly studied genera, where distinguishing characters remain to be identified.

By contrast, detailed studies to date have indicated that taxonomic data generated from DNA barcoding of individual organisms [19,20] or bulk environmental DNA metabarcoding of macroinvertebrate benthic samples provides a more complete snapshot of the range of taxonomic diversity present in the sample [21,22]. Moreover, if consistent extraction, amplification, and bioinformatics methods are applied, the results are also more repeatable and spatially consistent. This increases the reliability of the biodiversity signal while reducing the noise of inconsistent observation arising from taxa that cannot be identified (pseudoabsence), or which are present in the whole sample, but absent from subsampled material (sampling error). These twin problems of identification have plagued visual taxonomic analysis for decades but can now be eliminated to provide a more consistent biodiversity signal. Similar results have also been obtained for marine benthos [23].

DNA-based observation methods are throwing the often unacknowledged flaws of traditional observational methods into sharp relief. However, the full potential of these methods remains unrealized. For example, identification of specimens to a Linnean taxonomic name requires that taxonomically verified specimens have been previously barcoded and that this barcode sequence information has been deposited in an accessible database. For many groups, barcode libraries are quite advanced [24], for others, less so [25]. However, it has been argued that the lack of a barcode library may not preclude use of the information for ecological purposes (see below). It has proved possible to assign taxonomic meaning to sequence data based on prior knowledge of sequences of related taxa [26], and such methods can only improve as database coverage increases.

## 4. Standard barcodes for metabarcoding

A *sine qua non* of DNA barcoding is the use of standardized genetic markers for species identification. In animals, mitochondrial cytochrome c oxidase 1 (COI) DNA barcodes have been the designated genetic marker [15]. Subsequent to the introduction of DNA barcoding for animal taxa, other genes have been selected for barcoding fungi (ITS; [27]), plants (*rbc*L/*mat*K; [28]) and protists (18S rRNA [29]). However, DNA barcodes selected for non-animal taxa may not provide the same level of resolution at species level as compared to animal barcodes. In prokaryotes, the 16S rRNA gene is most commonly used for taxon identification [30,31] and has been used in a large number of microbial studies including microbiome surveys [32,33].

The use of standardized DNA barcodes provides the possibility of accessing a large and growing reference sequence library, which can facilitate large-scale and robust biodiversity analyses. Several studies have shown the utility of DNA barcodes in NGS analysis of bulk environmental samples [21,22,34–37]. However, there has been a debate on whether standardized DNA barcodes are suitable for the analysis of bulk environmental samples in a DNA metabarcoding framework and the analysis of environmental DNA [38]. Advocates of non-barcode genes are mainly concerned with the utility of PCR primers used for amplifying genes from environmental DNA. For example, they argue that high levels of variability in the COI barcode across different taxonomic groups can make it impossible to use a universal PCR primer-set for targeting biodiversity in an environmental sample [38]. They recommend using non-barcode markers with more conserved primer-binding sites such as mitochondrial 16S or 12S rDNA in animals [38–40]. Another consideration is the DNA fragment size of a genetic marker. It has also been noted that smaller markers, such as the plant chloroplast *trn*L intron P6 loop, are more suitable for amplifying and sequencing the presumably degraded DNA from environmental samples [41,42].

**Figure 2.**
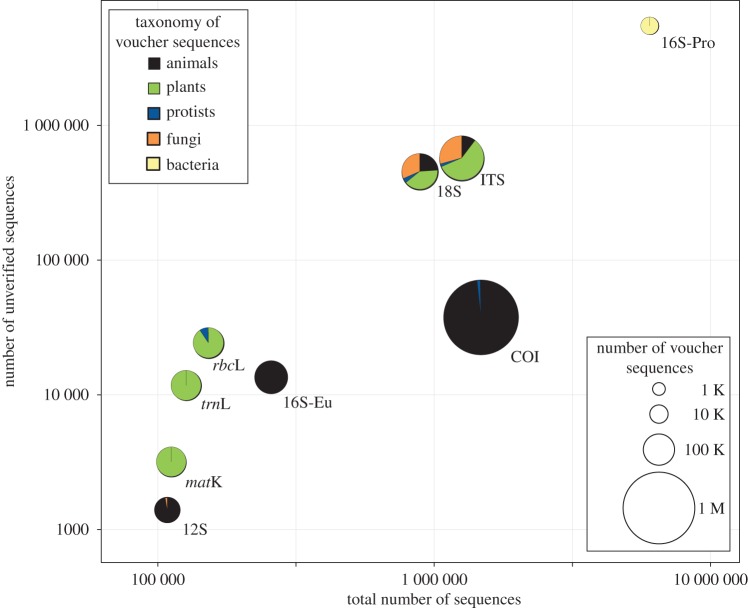
GenBank coverage of nine DNA markers commonly used in taxonomic identification. Number of sequence entries with unverified taxonomic identities (tagged with ‘unverified’, ‘environ*’, ‘uncultured’ or ‘clone’) is plotted against total number of sequences in the nucleotide database for each marker. Bubble size indicates the number of marker sequences with verified taxonomic identities (tagged with ‘verified’ or ‘voucher’) and colour shows relative taxonomic coverage of the verified sequences. 16S-Eu refers to mitochondrial 16S while 16S-Pro is the prokaryote 16S marker. All GenBank data were retrieved on 23 March 2016.

The use of non-barcode markers in environmental DNA metabarcoding comes with a significant cost. Most importantly, the vast and growing reference DNA barcode libraries cannot be used if non-barcode markers are sequenced (figure 2). Additionally, most of the non-barcode markers used in metabarcoding provide much lower taxonomic resolution as compared to standard DNA barcodes, which have been optimized for species-level analysis. For example, in a majority of cases the *trn*L intron P6 loop is uninformative for species- and genus-level identifications even when a reference database is available [41]. These factors can directly impact the utility of metabarcoding analysis in environmental biomonitoring programmes, where finer taxonomic resolution and access to a reference database for annotating environmental sequences are critical in developing biodiversity matrices for biomonitoring. It is our opinion that non-barcode markers are a useful tool in some specialized cases, but not for most aspects of biodiversity monitoring research where optimal data to gain better insights on species identity are crucial (e.g. rare or endangered species conservation, detection of invasive or pest species, presence of environmental quality–indicator species).

Owing to the critical importance of using DNA barcodes for biomonitoring and other ecological applications, especially in the light of advances in NGS technologies, the research community contributing to the Barcode of Life initiative and large-scale projects such as the International Barcode of Life (iBOL) have been working on optimizing protocols and using new technologies for DNA metabarcoding. A number of large-scale projects have specifically been launched to evaluate the use of DNA barcodes in biomonitoring through NGS analysis (see below). Past work has shown that optimal PCR amplification can be achieved through designing multiple primers and adding degeneracy to primer sequences to recover biodiversity in an environmental sample [34,43]. Additionally, genomics technologies such as whole-genome sequencing [44] or sequence capture [45] could provide an alternative to PCR amplification. Studies have provided insights on the use of standard DNA barcode markers for the analysis of samples with presumably degraded DNA such as gut contents [46] and aquatic environmental DNA [47].

## 5. From sequence reads to interpreting ecosystem change

Aside from hardware and processing capacity, a lack of optimized analytical paths or the expertise required for using high-performance computational tools can impede the application of DNA barcode data, especially in large-scale biomonitoring. In high-throughput DNA barcoding and metabarcoding analyses, the large volume of data creates challenges for data analysis including: dealing with embedded noise and inaccuracies in high-throughput sequencing (HTS) data; dealing with heterogeneous sequence data such as pseudogenes or contaminants; assigning sequences to units of biodiversity (e.g. species, especially when reference sequence data is lacking); visualizing and linking biodiversity data spatio-temporally; and statistical analysis of ecological change. Research and development is working to provide capacity to address some of the challenges in data analyses. For example, BOLD [48] is a globally recognized database and analysis platform for DNA barcode data, and GenGIS [49] is a highly efficient analysis environment for various relevant data analyses and visualization techniques. Tools and workflows developed for microbial ecology such as QIIME [50], MOTHUR [51] and the Ribosomal Database Classifier for taxonomic assignment [52] have been extremely successful, but they were specifically developed with the 16S gene in mind and their use requires a substantial amount of bioinformatics training. Although existing tools offer important models to build on, several aspects are in need of refinement before metabarcoding can gain widespread, routine use:

### (a) Specialized database construction

‘Biomonitoring-purposed’ databases can provide a higher degree of data quality while decreasing the database size. The LMAT and Kraken algorithms are a demonstration of the power that can be gained using new methods [53,54] combined with a well-structured database for species identification. DNA-based biomonitoring analyses can benefit from combining these k-mer approaches and homology-based approaches such as exact sequence matching, hidden Markov models and BLAST [55]. Furthermore, biodiversity inference can be improved by augmenting databases with sequences from type specimens, with uncharacterized taxa from environmental samples, with taxa known only from their DNA sequences, and with known indicator species that may signal various levels of pollutant effects on ecosystem health.

### (b) Taxonomic assignments

It is imperative to ensure the correct identification of taxa as a first step of subsequent analyses. Doing so will require specialized methods and databases, and improved methods for taxonomic assignment. When dealing with the large volumes of data generated by HTS, one of the most serious analysis bottlenecks occurs during the clustering of sequences to assemble diversity units. This step is done to increase efficiency, to mitigate the impacts of sequencing error, and to assemble conceptual units of diversity. The resulting operational taxonomic units or OTUs are often taken to serve as proxies for taxonomic units (e.g. species). Two widely used methods for cluster construction are UCLUST [56] and DNAClust [57]. Both methods rely on ‘centroid’ sequences that anchor clusters with a given degree of sequence similarity, but cluster boundaries are arbitrary and often dependent on the order in which sequences are clustered. Hybrid models and novel methods, such as those based on swarm dynamics [58,59], could improve the performance of these tools. Additionally, more rigorous assignment and delimitation techniques are being introduced to enhance taxonomic inference, especially at species level [60].

### (c) Phylogenetic and occupancy analyses

An alternative to taxon-based analysis is to compare observations phylogenetically. This approach avoids the need to make specific taxon calls. For example, a phylogenetic placement approach such as pplacer [61] uses a tree based on full-length reference genes and then maps short environmental sequences onto this tree based on the maximum-likelihood criterion. This approach can be applied to various taxonomic groups (including bioindicator assemblages). Another potentially valuable measure in biomonitoring is phylogenetic diversity, especially as it relates to ecosystem services [62]. Habitat occupancy modelling [63] can explicitly incorporate detection errors when using environmental DNA, and can provide a powerful method of estimating detection probability and occupancy rates as these approaches become more common [64]. Additionally, based on such an abundance–occupancy framework [65,66], one can examine the potential use of bulk DNA as a means to measure occupancy (presence/absence of taxa in sites), which can provide an indirect measure of relative abundance in a site or region.

## 6. Biomonitoring 2.0

**Figure 3.**
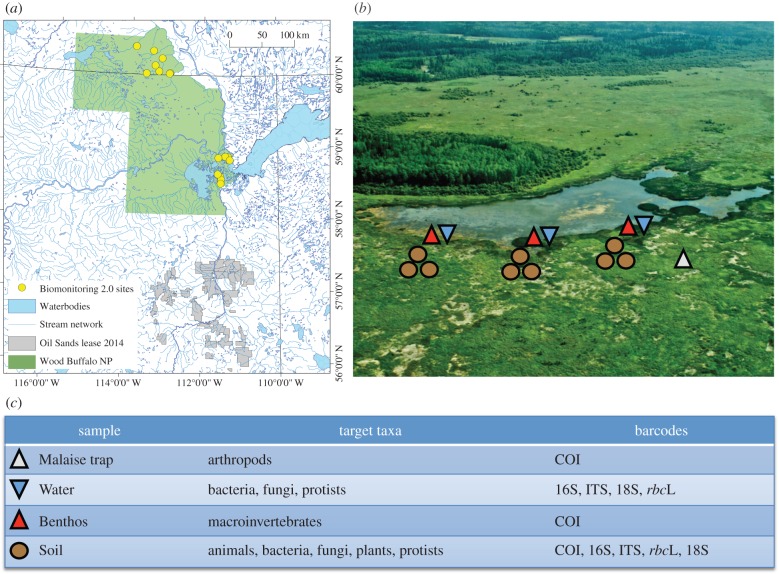
Biomonitoring 2.0, (*a*) a map of Wood Buffalo National Park, Alberta/NWT, indicating 16 sampling sites visited by the Biomonitoring 2.0 project from 2011 to 2014; (*b*) a photo of Egg Lake wetland, indicating spatial coverage of habitat samples collected from soil (brown circles), benthos (red triangles), water column (blue triangles) and malaise trap (white triangle); (*c*) DNA barcodes employed to capture biodiversity information from different habitat samples using metabarcoding.

Baird & Hajibabaei [67] proposed the term Biomonitoring 2.0 to describe a new way of thinking about ecosystem biomonitoring, a key component of which was the use of DNA barcodes generated through HTS as an integral biological data source. At that time, studies had demonstrated the potential of this new approach [21]. However, we suggested that a large-scale multi-habitat analyses would be required to assess the utility of metabarcoding from sampling to data analysis in a large-scale biomonitoring framework. A ‘Biomonitoring 2.0’ pilot project was subsequently carried out, with the first phase completed in 2015, focusing on wetland sites in Wood Buffalo National Park, which straddles the border between Alberta and the Northwest Territories in Canada's boreal region (figure 3). Although the sampling sites are within the boundaries of a protected national park, they are also downstream from the Alberta Oil Sands and, therefore, linked to an environmental assessment of downstream impacts. The primary objective of the project was to demonstrate and further develop the technical applicability of metabarcoding.

In the Biomonitoring 2.0 project, work focused on conventional benthos-based analysis as well as metabarcoding analyses of soil, water and Malaise trap (terrestrial arthropod) samples (figure 3). Samples were collected in two seasons over 4 years, and analysed to probe the biodiversity of prokaryotes and eukaryotes using their designated DNA barcode markers. In the case of benthic macroinvertebrates, the project involved comparing standard morphological data with metabarcoding data from a large number of bulk samples. Results to date clearly indicate enhanced spatial biodiversity resolution due to an increase in the information content from HTS DNA barcode data, both taxonomically and through ecological analysis based on sequence OTUs [22]. Our observations further support the use of complementary markers in the analysis of complex environmental samples to offset any gene or primer-specific bias in data generation [68].

The validation of metabarcoding in the Biomonitoring 2.0 project is now influencing other biomonitoring programmes and networks. For example, this approach was recently included in a major ecosystem-monitoring plan for the Canadian oil sands industry. Internationally, several initiatives are making use of DNA barcodes in biomonitoring applications such as the recently established Wetlands Ecosystem Genomics Analysis Network (WEGAN) [69]. These early examples provide clear evidence of a trend towards wider adoption of these technologies within the regulatory and industry sector, where there is a clear need to provide timely, science-based solutions in support of responsible resource development and sustainable management of vulnerable ecosystems.

## 7. Conclusion

DNA barcoding continues to demonstrate its disruptive potential as a tool to drive new thinking, support further testing of theory, and drive changes in the practice of ecosystem assessment. Technical advances are supporting and complementing new ways of thinking about taxonomy and phylogeny, leading to revolutionary views of biodiversity. Taxonomy has been rigorously applied since the time of Linnaeus, and yet it has adapted to new knowledge arising from scientific debate and new types of evidence [70,71]. DNA evidence is revolutionizing taxonomic practice, but it also highlights discrepancies between taxonomy, phylogeny and ecological characteristics. Given its emphasis on DNA evidence, metabarcoding presents a challenge to ecosystem scientists to enrich their taxonomy-based practices with biodiversity information in its broadest sense. Some have argued that the development of barcode libraries is an exercise in futility [72], given Wilson's challenge that biodiversity is ‘everything’ and the sheer audacity of attempting such a feat. While it is clear that we still have far to go before an ecosystem observation system that is capable of such broad-scale coverage can be implemented, the new biodiversity genomics tools described above are providing a solid platform on which to explore a more complete view of nature. The next challenge will be to harness this information to yield new insights into how ecosystems change by marrying the old Linnean traditionalist views with the emerging science generated from DNA-based biodiversity observation.
